# Predicting and Assessing Work Performance of People with Limited Work Capacity (LWC): A Multi-Wave, Multi-Source Study

**DOI:** 10.1007/s10926-020-09925-8

**Published:** 2020-09-10

**Authors:** Gemma M. C. van Ruitenbeek, Fred R. H. Zijlstra, Ute R. Hülsheger

**Affiliations:** 1 Department of Work and Social Psychology, Maastricht University, Maastricht, The Netherlands; 2 Department of Work and Social Psychology, Faculty of Psychology and Neuroscience, Maastricht University, P.O. Box 616, 6200 MD Maastricht, The Netherlands

**Keywords:** People with disabilities and limitations, Work behaviour, Task performance, Personal and professional development, Multi-source feedback

## Abstract

*Purpose* Occupational integration is vital for the health of all people, also for people with Limited Work Capacity (LWC). Therefore, participation in regular work is a legal right for people that are restricted in their work capacity due to a disability and/or lack sufficient education. Full and effective integration is dependent on the person-job fit, and adequate vocational support should focus on meeting performance standards, as is common practice in traditional personnel selection and development programmes. Despite the huge amount of valid instruments for personnel selection and development, these tests are not suitable people with LWC. Recently, an instrument was developed for assessment and development purposes specifically for this target group. That study provided evidence for reliability and dimensionality this instrument. In our study, we add criterion-related measures to this instrument to demonstrate that assessment at T1 predict performance at T2, thus validating the instrument. *Method* We conducted a four-source data study, two sources for independent and two for outcome variables, to test the predictive validity of this instrument in a multi-wave setup. *Results* This study largely supports the validity of the instrument in predicting work behaviour and task performance of people with LWC. More specific, when measures are tailored to this target group, this group is able to predict their work behaviour and task performance accurately just like the general population. *Conclusion* We conclude that this instrument contributes to science, vocational support practices, and the personal and professional development of people with LWC, which is required for sustainable work.

## Introduction

The psychological value of paid employment has been acknowledged for decades [1–3]. Employment is generally viewed to be conducive to mental health [4–6], whereas unemployment is associated with impaired mental health [6–10], with lower physical health [11], and with social isolation [12]. Employment should never be taken for granted, especially not for people with disabilities. The employment rate of people with disabilities remains far below that of people without limitations [13, 14], despite all public policies and legislations aimed at enabling workplace inclusion of people with disabilities [15]. We are referring to a group of people that has some kind of functional limitation. According to the International Classification of Functioning (ICF) [16], this is a rather diverse group which encompasses people with disabilities, but also people with low intellectual abilities, people with chronic diseases, and people with mental health issues that may vary from severe to ‘mild’ issues, such as attention deficit hyperactivity disorder (ADHD).

Participation in regular work of this target group is increased, but their employment is often of short duration [17, 18]. This is often caused by poor person-job fit [19], or poor guidance concerning learning and development on the job [20, 21]. Various approaches take the wishes and needs of specific target groups as point of departure in order to cover these concerns. Examples are the choose-get-keep approach [22], supported employment [23], and individual placement and support (IPS) [24], all for people with severe mental illness. Instruments that support methodical action of support providers of a broader group of people are lacking. Moreover, we argue that the performance standards in regular work should be the starting point, since the purpose is sustainable participation in regular work. Over decades, personnel psychologists have developed instruments that can facilitate the person-job fit and that can predict work performance [25–27], as these are important conditions for the duration of employment contracts. However, these instruments have been developed for the general population, and are not suitable for our target group. A person with functional illiteracy, for example, may be able to use familiar everyday expressions and very basic phrases, but is not able to fill-out complex questionnaires, and individuals with autistic spectrum disorders face difficulties with respect to metaphorical language often used in traditional personality questionnaires. Therefore, we need to design instruments with unambiguous and simple language.

In line with what the ICF model [16] assumes, human functioning is a result of a dynamic interaction between the limitation, personal characteristics and external factors. The dynamic interaction means that interventions directed at one aspect can cause changes in other related aspects. We therefore, plead for an instrument that includes both personal characteristics (such as coping style) and external factors (such as performance standards) in order to determine the direction of guidance.

**Fig. 1:**
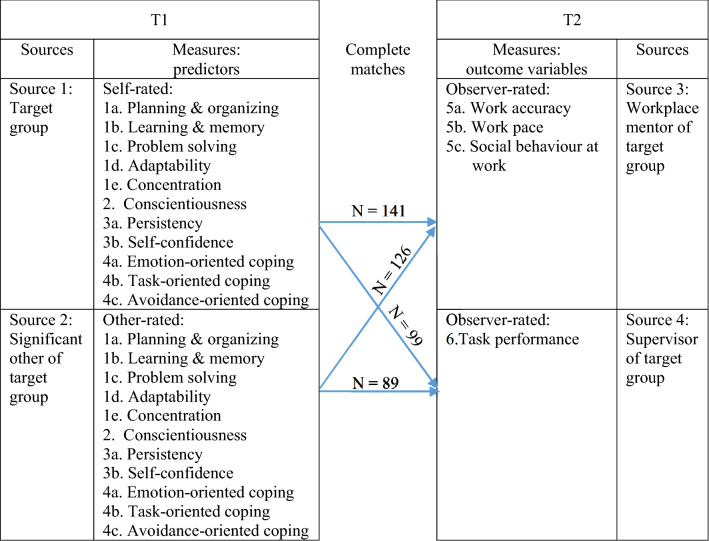
Study setup and sample size

Recently, Van Ruitenbeek et al. [28] have developed the Maastricht Work Capacity Monitor (MW©M) in an endeavour to facilitate selection, placement, and development of individuals with LWC, individuals like people of our target group. Although they provided evidence for the reliability and construct validity, the predictive validity has remained unaddressed. The overall goal of the present study is twofold. First, we will evaluate the predictive validity of the MW©M on performance standards such as work behaviour outcomes of our target group using a rigorous multi-source study in a longitudinal set-up (see Fig. 1). Second, we will extend the MW©M with measures of work behaviour and task performance that we adjusted to our target group.

In doing so, we make the following contributions to the literature: first and foremost, we draw attention to an important and yet understudied population that has the legal right to work and participate in the labour market but that has largely been ignored in the work and organisational psychology literature (for a recent exception see Vornholt et al. 2018) [15]. This is problematic, as organisations that are willing to employ people from the target group have insufficient means to select, place and train these people within their organisation because traditional personnel selection and development instruments are not designed for this specific population. We contribute to the work and organisational psychological literature as well as to the occupational rehabilitation literature, which will benefit from the availability of an adjusted and validated instrument to study the relationships between personality traits and work behaviour of people with LWC more accurately. Second, we expect that with our supplements, the MW©M can contribute to practice as it can enhance adequate assessment of the work capacity (personnel selection and matching person-job fit).We expect that it can contribute methodically to the continuous monitoring principle of supported employment interventions, such as IPS [29]. Third, we expect that our contribution will empower the field of work and organisational psychology and human resources practices to include people with LWC in paid employment.

Finally yet importantly, our target group can learn to reflect on their own strengths and weaknesses with the help of this instrument that consists of self-, other-, and observer-ratings forms. This helps to get an understanding of one’s own work behaviour that can serve future behaviour, as an important driving force for learning [30–32]. This strengthens their personal and professional development, and sustainable integration into work.

## Point of Departure; Assessing Performance Standards of People with LWC

As stated in the introduction, we argue that performance standards in regular work should be the point of departure when the purpose of our target group’s sustainable participation in regular work. These standards are conditional for the continuation of their employment contract. There is a call for adequate and fair assessment instruments [33], and for more narrow and job-focused measures of performance that can be used for formal job appraisals as well as providing feedback to employees [33]. We argue that the task performance scale of Williams and Anderson [34] fits these criteria. This task performance scale also focuses on important work aspects such as quality and efficiency. We therefore adapted the task performance measure of Williams and Anderson [34] to reflect performance requirements for individuals with LWC.

Nearly every job, from cleaning services to ICT services, is performed in a customer or client service driven organisation nowadays. People need to display certain behaviour at work in order to be able to deliver the expected performance [35]. For that reason, we measure aspects of work behaviour itself that are relevant and expected to be displayed as part of every job alongside task performance. We argue that work behaviour and functioning should be measured adequately and fairly [36] for this specific population. The work behaviour inventory (WBI) [37] is an adequate instrument, that was developed for people with severe mental illness. We adapted the scale in order to make the scale more suitable for people with common mental disorders and disabilities.

The specific properties of the work behaviour and the task performance scales that we used in this study are described in the method section.

## Predictors of Work Behaviour of LWC

An important question that has kept psychologists busy over the years is: what predicts work behaviour for the general population [25–27]? Special attention for predictors of work behaviour for people with LWC was seldom given. Van Ruitenbeek et al. [28] deliberately selected personal characteristics and personality traits relevant to the work capacity of individuals with LWC, and developed the MW©M. In choosing personality traits, they built upon extensive evidence from the work and organisational psychology literature and combined this with insights from the occupational rehabilitation literature. In this section, we complement their work and revisit the theoretical rationale for the choice of predictor variables included in the MW©M: mental ability, conscientiousness, self-efficacy and coping.

Several studies consider *mental ability* to be the most valid predictor of job behaviour [27, 38, 39]. General mental ability or intelligence reflects individuals’ capability to reason logically, solve problems, make decisions, think abstractly, and learn [27, 38]. Nevertheless, the type of work and its context define the importance of mental ability [39], meaning that the required level of mental ability depends on the level of job complexity. Although logical reasoning and sophisticated problem solving skills are relevant for complex jobs, simple or routinized tasks require basic mental ability skills. In line with Van Ruitenbeek et al. [28], we argue that basic cognitive skills, such as planning and organising, learning and memory, adaptability, concentration, and basic problem solving, are important predictors of work behaviour for people with LWC. These aspects cover the key cognitive elements that can affect the ability to function in the workplace, and are assumed to be important aspects to include in assessment of work-capability [40]. Based on these considerations, we expect these five dimensions measured by self-rating and other-rating scales for people with LWC to be positively related to work behaviour and task performance.

*Conscientiousness* is one of the ‘big five’ personality traits that can be seen as a second powerful predictor of work behaviour [25–27]. Conscientious individuals are characterized by a high orientation to accomplish tasks, trustworthiness and cautiousness [25, 41, 42]. Among the ‘big five’ traits, conscientiousness is the personality trait with the strongest predictive validity across different kinds of jobs and occupations including lower-level jobs [42]. We therefore argue, in line with Van Ruitenbeek et al. [28], that conscientiousness measured by self-rating and other-rating scales for people with LWC will be positively related to work behaviour and task performance.

The validity of s*elf*-*efficacy* has been demonstrated by several authors [43, 44]. Self-efficacy can be seen as the tendency to believe in one’s own capacities and effectiveness to meet work demands in a variety of work settings [44–46]. Moreover, self-efficacy stipulates the course of action, the level of effort people put in, and their persistency [45, 47, 48]. It represents characteristics that demonstrated predictive validity to work-related behaviour for each level of task complexity across all included studies in a meta-analysis conducted by Stajkovic and Luthans [44]. The scales for people with LWC measure personal characteristics, such as self-confidence and persistence. These personal characteristics are important attributes for accomplishing work tasks [28]. In line with this reasoning, we expect that persistency and self-confidence measured by self-rating and other-rating scales for people with LWC will be positively related to work behaviour and task performance.

Finally, several authors consider self-regulation strategies to be important predictors of work behaviour [24, 49–51]. *Coping* can be seen as subset of self-regulation [52]. Coping refers to mental and behavioural effort people expose in order to master or decrease effects of stressors [53, 54]. Coping also reduces the impact of any kind of limitation or problem individuals face [55] as a consequence of their disability or disorder. Previous research has provided evidence for the positive relationship between self-regulation or self-management skills and work behaviour [49]. Although these studies have been conducted in the general population, Van Ruitenbeek et al. [28] argue that coping is an important predictor of work performance especially for people with LWC, since they have to deal with serious restrictions due to their functional limitation. The coping scale for people with LWC [28] is based on the shortened coping inventory for stressful situations (CISS-21) [56]. This three-factor structured coping scale refers to emotion-oriented coping, task-oriented coping, and avoidance-oriented coping. This model is grounded in the conceptualisation of coping as deliberate responses aimed at: (a) reducing stress through emotional reactions (emotion-oriented coping), or (b) solving the problem and attempting to change the situation (problem-focussed or task-oriented coping), or (c) seeking support and protection from others [57] and avoiding the stressful situation through distracting oneself with other situations or tasks (avoidance-oriented coping) [56]. In general, problem-focussed or active coping (i.e. task-oriented coping) has been shown to correlate with better adjustment, whereas emotion-oriented coping and withdrawal (avoidance-oriented coping strategies) have been found to be associated with poorer adjustment [55, 57]. Therefore, we expect task-oriented coping to be positively related to work behaviour and task performance, whereas we expect emotion-oriented and avoidance-oriented coping strategies to be negatively related to work behaviour and task performance.

Taken together, the arguments presented above suggest that mental ability, conscientiousness, self-efficacy and coping assessed by self- and other-ratings at time 1 predict work behaviour and task performance at time 2. We therefore hypothesise:

### H1

*The self*-*rating form* of the MW©M measured at T1, consisting of scales for planning and organising, learning and memory, problem solving, adaptability, concentration, persistency, self-confidence, emotion-oriented coping, task-oriented coping and avoidance-oriented coping, predicts both (a) work behaviour and (b) task performance measured at T2.

### H2

*The other*-*rating form* of the MW©M measured at T1, consisting of scales for planning and organising, learning and memory, problem solving, adaptability, concentration, persistency, self-confidence, emotion-oriented coping, task-oriented coping and avoidance-oriented coping, predicts both (a) work behaviour and (b) task performance measured at T2.

## Method

We collected data from four different sources (i.e., target persons, significant others, workplace mentors and supervisors) in a multi-wave design in order to test the predictive validity of the MW©M-scales [28] on work behaviour and task performance. Specifically, we used four different sources of data: self- and other-ratings of independent variables (MW©M-scales for mental ability, conscientiousness, self-efficacy and coping) measured at time 1, and ratings of the workplace mentor and the supervisor of outcome measures (such as work behaviour and task performance) at time 2 (see Fig. 1). People from the target group and their significant other (individuals that were chosen by the target persons themselves and who know the target person well, such as relatives or personal coaches) completed the self- and other-rating form of independent variables just before or in the first couple of days of work. After approximately 4 weeks of work, a workplace mentor completed a questionnaire assessing work behaviour, and the supervisor completed a questionnaire assessing task performance. The time span of approximately 4 weeks of work allows the target person to familiarise him- or herself with the work and work context and provides mentors, colleagues and supervisor with the opportunity to get to know the target person and his or her work behaviour and task performance. This time span also allows professionals to give feedback to our target group about their work behaviour and task performance in the first couple of workweeks in case a change in behaviour is needed. We deliberately chose the perspective of both mentors and supervisors, because the workplace mentor observes of the target person’s day-to-day work behaviour and interacts with them, while the supervisor has more insights into work outcomes and performance levels.

We recruited participants from three vocational support providers in the Netherlands and the Dutch Employee Insurance Agency (Uitvoeringsinstituut Werknemersverzekeringen, abbreviated UWV). We collected data between November 2014 and February 2018.

### Procedure

We contacted several vocational support providers and the UWV who offer vocational rehabilitation for people of our target group, and we presented the outline of the research project. Seven agencies with interest in collaborating received detailed information about the study and we asked job coaches and rehabilitation consultants to coordinate the participation of people from the target group. These coordinators registered the participants online in order to receive a registration code that enabled the target person, their significant other, their workplace mentor and the supervisor to fill in the online questionnaires. Prior to the questionnaires, participants were informed about the procedure and their rights with respect to the research in the online survey. The coordinator guided this process, which included, if necessary, the direct guidance of our target group when they filled-out the online questionnaire. Only fully accountable participants were included. They signed the online informed consent themselves. At time point one, the link to the online questionnaires for the self-rating and other-rating were distributed by e-mail. Four weeks after the target person had started working (time point two), workplace mentors and supervisors received online invitations to fill in the work behaviour and work outcome scales, respectively. Participants obtained a report of their individual results when the questionnaires were completed. The study has been approved by the local ethical review board.

### Participants

People from the target group (*N* = 267) and their significant other (*N* = 199) completed a questionnaire that consisted of mental ability, conscientiousness, self-efficacy and coping scales. On average after 5.3 weeks of the target person starting to work, a workplace mentor completed the work behaviour questionnaire and the supervisor completed the task performance questionnaire. This resulted in complete matches for the self- and other-rated predictors and observer-rated work behaviour, and task performance of *N* = 141, 126, 99, and 89 respectively.

The self-report sample of complete matches of self-report (T1) and observer-report (T2) consisted of 141 self-ratings (64.5% male, see Table 1). The mean age of the participants was 28.95 (*SD* = 10.02). More than three-quarters of the target group dealt with disabilities varying from a learning disability (25.9%), AD(H)D or autism spectrum disorders (24.5%), psychological disorder (15.8%), physical (7.2%) or other (3.6%; such as brain injury or dyslexia). Almost a quarter (23%) of the target group did not report a disability. More than twenty percent (21.9%) of the respondents reported comorbidity. Several people faced additional personal problems such as debts (7.1%), housing problems (2.4%), problems with childcare (0.8%) or other personal problems (3.1%; such as dealing with grief or a disabled partner in combination with one’s own disability). The level of education varied from lack of or insufficient vocational education (72.3%), to low-level vocational education (13.9%), intermediate or secondary level vocational education (11.7%), and to high level education (2.2%). More than ten percent (11.9%) of the respondents had an employment contract, 32.2 percent had a learn-work agreement, 25.4 percent performed voluntary work, 25.4 percent worked in sheltered workplaces, and 5.1 percent worked during an internship. The work of the participants varied from simple duties in the care, service, or construction sector, to more complex administrative jobs.

**Table 1:** Descriptive statistics about the study population and the relation to their significant other

	Self-rated sample (N=141)				Significant other sample (N=126)	
	Mean	*SD*	Range	Missing	Percentage	Missing
Age (years)	28.95	10.02	16–58			
Gender (% men)	64.5					
Disability in percentages				2		
Not specified	23.0					
Learning disability	25.9					
AD(H)D/Autism spectrum disorder	24.5					
Psychological	15.8					
Physical	7.2					
Different	3.6					
Comorbidity	21.9					
Problems				14		
Housing	2.4					
Childcare	0.8					
Debts	7.1					
Different (not specified)	3.1					
Level of vocational education				4		
Lack or insufficient	72.3					
Low	13.9					
Secondary	11.7					
High	2.2					
Type of contract				23		
Regular contract	11.9					
Learn-work agreement	32.2					
Voluntary work	25.4					
Probation period/internship	5.1					
Sheltered workplace	25.4					
Branch/type of industry				20		
Administration	11.3					
Cleaning	3.5					
Care	12.2					
Catering	12.2					
Facilities	20.0					
Logistics	4.3					
Production	25.2					
Retail	5.2					
Information and communication technology (ICT)/media	5.2					
Different	0.9					
Relation to significant other						1
Parent					31.2	
Partner					5.6	
Family member					8.8	
Friend					10.4	
Job-coach					10.4	
Personal coach					29.6	
Workplace mentor					4.0	

The other-rating sample of complete matches of significant other (T1) and observer-report (T2) consisted of 126 other-ratings. Their relation to the target group varied from parent (31.2%), partner (5.6%), family member (8.8%), friend (10.4%), job coach (10.4%), and personal coach (29.6%) to a workplace mentor (4%).

### Measures

Mental ability was measured with the Vocational Cognitive Ratings Scale of Greig and colleagues [58], as adapted by Van Ruitenbeek et al. [28], consisting of five subscales: planning and organising, learning and memory, problem solving, adaptability, and concentration (e.g. “I complete tasks in a logical order.”, “I can remember well how to do something.”, or “I am easily distracted”).^1^

Conscientiousness was measured with the Dutch HEXACO personality inventory of De Vries et al. [59], as adapted by Van Ruitenbeek et al. [28], consisting of nine items (e.g. “If I have to do something, I prepare it.” or “I think carefully before making a decision.”).

Self-efficacy was measured with the general self-efficacy scale (GSES-12) of Bosscher and Smit [45], as adapted by Van Ruitenbeek et al. [28], that consisted of two subscales: (a) persistency (six items, e.g. “Even if I don’t like a task, I keep working on it until I’m done.”) and (b) self-confidence (four items, e.g. “When I really want to do something, it goes wrong.”).

The coping scale of Calsbeek and collegues (CISS-21) [56], as adapted by Van Ruitenbeek et al. [28], consisted of three subscales: (a) emotion-oriented coping (seven items, e.g. “If I have a problem or feel stressed, I blame myself for getting into that situation.”), (b) task-oriented coping (six items, e.g. “If I have a problem or feel stressed, I try to remember if I have had the same problem before and how I solved it.”, or “If I have a problem or feel stressed, I ponder if I can learn from it.”), and (c) avoidance-oriented coping (three items, e.g. “If I have a problem or feel stressed, I buy something for myself.”).

Work behaviour was measured by a scale that was inspired by the WBI [37]. The WBI was developed for people with severe mental illness. We translated the original English scale into Dutch, and adapted the scale in order to make it more suitable for people with common mental disorders and disabilities. We dropped items that were specifically related to severe mental illnesses (e.g. “Does not appear overly distant or aloof” or “Does not become overexcited or aggressive”). We also dropped items that reflect mental ability because they overlap with the independent mental ability scale. Before using the scale, supported employment experts verified the suitability of the scale for our target group and its relevance and applicability to the work situation. Given the changes we made to the original WBI scale, we tested the factor structure of the adapted scale. We subjected the data to exploratory factor analysis (EFA) using SPSS version 24. This process yielded a three-factor solution measuring, (a) work accuracy (seven items, e.g. “Arrives on time.” and “Works precisely.”), (b) work pace (four items, e.g. “Can keep up the work pace.” and “When he/she has to work faster, he/she makes mistakes.”), and (c) social behaviour at work (10 items, e.g. “Pays attention when listening to others.” and “Consults with the person he/she works with.”). We conducted a series of confirmatory factor analyses (CFA) and tested alternative models using M*plus* version 7.3 following procedures recommended in literature [60, 61]. Specifically, we tested a one-factor model with all items loading onto one factor (χ^2^ = 814.262, *df* = 189, *p* < .000; CFI = .649; TLI = .610; RMSEA = .151). We also tested this three-factor model with work accuracy loading on one, work pace on another and social behaviour at work loading on a third factor. Examination of fit indices indicated a poor fit for the work behaviour scale: *χ*^2^ = 530.395, *df* = 186, *p* =.000, CFI = .807, TLI = .785, RMSEA = .113. SRMR = .080 (*N* = 146). Inspection of the modification indices indicated that a better fit could be obtained by inclusion of four residual covariances. We accepted residual covariances because items were largely similar but at the same time, they indicated important subtle differences (‘Arrives on time.” and “Is present am at work as agreed.”; “I work precisely” and “Makes sure he/she don’t skip anything”; “Takes care of his/her appearance” and “I adjust my clothing to the work”; and finally “Works more slowly than others” and “When he/she has to work faster, he/she makes mistakes”). The model fit indices improved substantially after this adaptation: *χ*^2^ = 328.333, *df* = 182, *p* = .000, CFI = .918, TLI = .905 RMSEA = .074, SRMR = .068. We therefore treated work behaviour as a three-dimensional construct in this study.

For task performance, we adapted the task performance scale of Williams and Anderson [34]. We re-worded a few items from this original task performance scale in order to make it more applicable to the context of the target group. For example, we used “The work is done on time.” instead of “Engages in activities that will directly affect his/her performance evaluation”.

All scales were answered on a five-point Likert scale: 1 = never, 2 = almost never, 3 = regularly, 4 = almost always, 5 = always.

### Analysis

In order to explore the criterion-related validity of the self-ratings and other-ratings of mental ability, conscientiousness, self-efficacy and coping on work behaviour and task performance, we first inspected zero-order correlations between the independent and dependent variables separately for each rating source.

Next, we conducted multiple regression analyses separately for the self- and other-ratings of independent variables using SPSS version 25. Since the traditional multiple regression approach has been criticized not to partition variance appropriately between various predictor variables, we also conducted relative weight analyses (RWA) [62], as it enabled us to test the relative importance of variables [63] using RWA-Web [64]. As recommended by Tonidandel and LeBreton [64], confidence intervals for the individual relative weights [65] and all corresponding significance tests were based on bootstrapping with 10,000 replications. Accelerated confidence intervals were used because of their superior coverage accuracy.

Before testing our hypothesis, we conducted a set of preliminary analyses to ensure that no violations of the assumptions of normality and linearity were made. We examined the critical Chi square values for evaluating Mahalanobis distance on outliers by using the number of independent (11) variables as the degrees of freedom [66, 67]. Results indicated three outliers with critical Chi square values above 31.26 in the self-rating sample. After deletion of these three cases, the final sample consisted of 138 participants.

## Results

As can be seen from Table 2 (results of self-ratings) and Table 3 (results of other-ratings), results showed relatively low alphas for some independent variables. We therefore checked for the mean inter-item correlation for scales with alpha’s below .7. The mean inter-item correlations of adaptability, conscientiousness and avoidance-oriented coping amounted respectively .35, .22 and .32. As optimal mean inter-item correlation ranges from .2 to .4 [68]., all three fit these criteria.

**Table 2:** Descriptive statistics and intercorrelations between study variables of self-rating

	Correlations														
	1a.	1b.	1c.	1d.	1e.	2.	3a.	3b.	4a.	4b.	4c.	5a.	5b.	5c.	6.
1a. Planning and organizing	–														
1b. Learning and memory	.59**	–													
1c. Problem solving	.38**	.38**	–												
1d. Adaptability	.20*	.25**	.24**	–											
1e. Concentration	.43**	.32**	.14	.48**	–										
2. Conscientiousness	.68**	.45**	.37**	.06	.25**	–									
3a. Persistency	.69**	.51**	.34**	.20*	.42**	.61**	–								
3b. Self-confidence	.32**	.22**	.14	.51**	.45**	.18*	.23**	–							
4a. Emotion-oriented coping	.01	− .09	− .05	− .28**	− .23**	.06	− .05	− .60**	–						
4b. Task-oriented coping	.38**	.25**	.43**	.18*	.30**	.35**	.51**	.24**	− .07						
4c. Avoidance-oriented coping	.10	.10	.10	.13	− .02	.07	.08	.01	.04	.27**	–				
5a. Work accuracy	.25**	.08	− .04	.05	.19*	.16	.25**	− .03	.11	.06	− .15	–			
5b. Work pace	.21*	.23**	.11	.20*	.22**	.12	.23**	.15	− .22*	.14	− .13	.50**	−		
5c. Social behaviour at work	.27**	.10	.01	.06	.19*	.23**	.37**	.11	.03	.14	− .16	.73**	.54**	–	
6. Task performance	.16	.06	− .09	.06	.25*	.16	.27**	.05	.07	.10	− .23*	.57**	.45**	.50**	–
*n*	138	138	138	138	138	138	138	138	138	138	137	138	138	138	99
*M*	27.29	15.61	6.22	7.45	7.57	30.12	22.53	14.72	17.21	19.33	6.09	28.97	14.63	38.65	27.04
*SD*	5.07	2.83	2.03	1.82	1.89	4.53	4.34	3.15	6.33	5.25	2.61	5.10	3.67	7.02	5.69
*α*	.84	.77	.78	.51	.78	.69	.75	.74	.85	.84	.59	.87	.88	.87	.93

*P < .05 **P < .01

**Table 3:** Descriptive statistics and intercorrelations between study variables of the other-rating

	Correlations														
	1a.	1b.	1c.	1d.	1e.	2.	3a.	3b.	4a.	4b.	4c.	5a.	5b.	5c.	6.
1a. Planning and organizing	–														
1b. Learning and memory	.66**	–													
1c. Problem solving	.66**	.58**	–												
1d. Adaptability	.46**	.36**	.47**	–											
1e. Concentration	.63**	.42**	.40**	.55**	–										
2. Conscientiousness	.78**	.58**	.52**	.28**	.51**	–									
3a. Persistency	.80**	.50**	.52**	.43**	.50**	.75**	–								
3b. Self-confidence	.58**	.38**	.53**	.62**	.58**	.48**	.62**	–							
4a. Emotion-oriented coping	− .30**	− .19*	− .23*	− .49**	− .42**	− .16	− .27**	− .55**	–						
4b. Task-oriented coping	.68**	.50**	.56**	.42**	.41**	.65**	.65**	.53**	− .25**	–					
4c. Avoidance-oriented coping	− .36**	− .20*	− .34**	− .44**	− .28**	− .31**	− .44**	− .56**	.52**	− .41**	–				
5a. Work accuracy	.20*	− .03	.06	.05	.27**	.30**	.24**	.01	.04	.12	.04	–			
5b. Work pace	.21*	.14	.25**	.33**	.22*	.20*	.24**	.25**	− .29**	.18*	− .22*	.49**	–		
5c. Social behaviour at work	.25**	.05	.15	.23**	.22*	.29**	.30**	.21*	− .12	.25**	− .15	.71**	.57**	–	
6. Task performance	.25*	.10	.11	.20	.29**	.30**	.25*	.17	− .13	.18	− .12	.57**	.48**	.51**	–
*n*	124	125	124	124	123	125	126	125	126	126	126	126	126	126	89
*M*	25.23	14.94	6.00	7.27	7.38	29.03	21.10	14.37	4.81	9.21	5.30	28.97	14.65	38.69	26.67
*SD*	6.12	2.99	2.02	1.82	2.06	6.30	5.14	2.93	1.93	3.11	2.15	5.02	3.71	6.69	5.68
*α*	.90	.83	.85	.76	.78	.89	.87	.72	.74	.85	.75	.87	.87	.86	.93

*P < .05 **P < .01

The dimensions concentration and persistency were positively related to all outcome variables in both samples. This applies also to the relation between planning and organising and the outcome variables, except for the non-significant relation between planning and organising and task performance in the self-report sample. In the other-rating sample, conscientiousness was positively related to all outcome variables as well, whereas in the self-report, there was only a positive relation between conscientiousness and social behaviour at work.

In the self-report, only the positive relation between conscientiousness and social behaviour at work was significant. While no significant relation of learning and memory was found in the other-ratings, learning and memory was positively related to work pace in the self-report sample. Problem solving, on the other hand, correlated positively with work pace in the other-ratings, whereas no significant relation was found between self-rated problem solving and any outcome variable. Adaptability and emotion-oriented coping showed positive relations with work pace in both self-rating and other-rating forms. Task-oriented coping showed only significant and positive relations in other-ratings. Only avoidance-oriented coping showed a negative relation with task performance. Self-confidence showed significant and positive relations with work pace and social behaviour at work in the other-rating sample.

Results of multiple regression and relative weight analyses are reported together in Table 4 (self-rating) and Table 5 (other-rating). When considered jointly in a multiple regression analysis, self-ratings of all 11 dimensions explained between 16% (work accuracy), 17% (work pace), and 20% (social behaviour at work) of variance in the respective aspects of work behaviour and 21% of variance in task performance. Owing to the intercorrelations between the 11 predictor dimensions, many individual beta-coefficients were not significant. A notable exception is self-reported avoidance-oriented coping, which was a significant negative predictor of all 4 outcome measures. Emotion-oriented coping was a significant negative predictor of work pace in multiple regression. Overall, multiple regressions regarding the self-rating report partly confirmed H1.

**Table 4:** Summary of multiple regression and relative weight analysis of the self-rating form

Predictor	*β*^a^	RW^b^	CI-L^b^	CI-U^b^	RS-RW (%)^b^
Criterion = work accuracy (R^2^= .162; F[11,125] = .912, p < .018^a^)					
1a. Planning and organizing	.26	.036	− .007	.105	22.26
1b. Learning and memory	− .10	.005	− .068	.015	3.07
1c. Problem solving	− .15	.010	− .034	.060	6.30
1d. Adaptability	.10	.004	− .060	.022	2.66
1e. Concentration	.10	.018	− .022	.088	11.11
2. Conscientiousness	− .02	.010	− .038	.037	6.35
3a. Persistency	.16	.031	− .008	.088	19.11
3b. Self-confidence	− .16	.008	− .045	.038	5.17
4a. Emotion-oriented coping	.06	.009	− .042	.051	5.78
4b. Task-oriented coping	.02	.004	− .062	.015	2.44
4c. Avoidance-oriented coping	− .18*	.026	− .012	.088	15.75
Criterion = work pace (R^2^= .165; F[11,125] = .908, p < .016^a^)					
1a. Planning and organizing	.15	.015	− .008	.070	9.19
1b. Learning and memory	.11	.020	− .006	.081	12.00
1c. Problem solving	− .05	.002	− .025	.025	1.25
1d. Adaptability	.16	.020	− .007	.088	12.16
1e. Concentration	.04	.014	− .008	.076	8.20
2. Conscientiousness	− .04	.004	− .025	.020	2.25
3a. Persistency	.07	.016	− .010	.067	9.35
3b. Self-confidence	− .20	.006	− .019	.026	3.73
4a. Emotion-oriented coping	− .25*	.036	− .004	.145	21.84
4b. Task-oriented coping	.10	.008	− .011	.057	5.04
4c. Avoidance-oriented coping	− .19*	.025	− .006	.129	14.99
Criterion = social behaviour at work (R^2^= .204; F[11,125] = .866, p < .002^a^)					
1a. Planning and organizing	.08	.027	− .031	.071	13.19
1b. Learning and memory	− .12	.007	− .065	.016	3.27
1c. Problem solving	− .11	.006	− .062	.020	3.02
1d. Adaptability	.03	.002	− .069	.012	.90
1e. Concentration	.01	.011	− .048	.043	5.25
2. Conscientiousness	.01	.018	− .046	.039	8.88
3a. Persistency	.39**	.083*	.009	.160	40.91
3b. Self-confidence	.07	.005	− .063	.020	2.50
4a. Emotion-oriented coping	.09	.004	− .066	.025	1.82
4b. Task-oriented coping	.01	.010	− .057	.030	4.90
4c. Avoidance-oriented coping	− .19*	.031	− .021	.105	15.36
Criterion = Task performance (R^2^= .210; F[11,86] = .906, p < .030^a^)					
1a. Planning and organizing	− .04	.009	− .078	.030	4.45
1b. Learning and memory	− .05	.004	− .091	.019	1.83
1c. Problem solving	− .21	.022	− .031	.112	10.67
1d. Adaptability	.06	.005	− .074	.029	2.25
1e. Concentration	.18	.035	− .016	.127	17.50
2. Conscientiousness	.07	.011	− .057	.039	5.40
3a. Persistency	.26	.042	− .026	.132	19.99
3b. Self-confidence	− .02	.003	− .089	.015	1.32
4a. Emotion-oriented coping	.13	.009	− .044	.063	4.27
4b. Task-oriented coping	.08	.010	− .059	.048	4.69
4c. Avoidance-oriented coping	− .26*	.058	− .025	.190	27.63

*p < .05 **p < .01

^a^Results from multiple regression analysis using SPSS version 25

^b^Results form relative weight analysis using R

*b* unstandardized regression weight, *β* standardized regression weight, RW raw relative weight (within rounding error raw weights will sum to R^2^), CI-L lower bound of confidence interval used to test the statistical significance of raw weight, CI-U upper bound of confidence interval used to test the statistical significance of raw weight, RS-RW relative weight rescaled as a percentage of predicted variance in the criterion variable attributed to each predictor (within rounding error rescaled weights sum to 100%)

**Table 5:** Summary of multiple regression and relative weight analysis of other-ratings

Predictor	*β*^a^	RW^b^	CI-L^b^	CI-U^b^	RS-RW (%)^b^
Criterion = work accuracy (R^2^= .247; F[11,109] = .829, p < .001^a^)					
1a. Planning and organizing	− .10	.020	− .007	.043	8.23
1b. Learning and memory	− .34**	.030*	.001	.108	12.32
1c. Problem solving	.06	.006	− .023	.033	2.42
1d. Adaptability	.05	.006	− .024	.026	2.30
1e. Concentration	.33*	.055*	.006	.143	22.08
2. Conscientiousness	.37*	.057*	.013	.128	22.87
3a. Persistency	.26	.034*	.003	.087	13.76
3b. Self-confidence	− .31*	.017	− .006	.070	6.84
4a. Emotion-oriented coping	.02	.005	− .018	.043	2.08
4b. Task-oriented coping	− .03	.009	− .018	.034	3.72
4c. Avoidance-oriented coping	.11	.008	− .011	.060	3.38
Criterion = work pace (R^2^= .165; F[11,109] = .919, p < .039^a^)					
1a. Planning and organizing	− .17	.006	− .036	.019	3.65
1b. Learning and memory	− .07	.003	− .037	.020	1.85
1c. Problem solving	.17	.019	− .011	.087	11.71
1d. Adaptability	.24	.043*	.003	.121	25.97
1e. Concentration	− .01	.008	− .015	.044	4.95
2. Conscientiousness	.18	.0111	− .014	.056	6.73
3a. Persistency	.15	.013	− .013	.059	7.75
3b. Self-confidence	− .13	.009	− .030	.030	5.37
4a. Emotion-oriented coping	− .22	.039	− .001	.126	23.63
4b. Task-oriented coping	− .06	.004	− .034	.019	2.49
4c. Avoidance-oriented coping	.01	.010	− .014	.058	5.89
Criterion = social behaviour at work (R^2^= .159; F[11,109] = .926, p < .051^a^)					
1a. Planning and organizing	− .02	.015	− .040	.039	9.32
1b. Learning and memory	− .26*	.016	− .017	.082	9.97
1c. Problem solving	− .01	.005	− .061	.018	3.22
1d. Adaptability	.20	.022	− .019	.094	13.85
1e. Concentration	.03	.011	− .031	.052	6.70
2. Conscientiousness	.27	.034	− .013	.097	21.62
3a. Persistency	.15	.026	− .017	.077	16.52
3b. Self-confidence	− .08	.007	− .058	.025	4.09
4a. Emotion-oriented coping	− .03	.003	− .048	.023	1.97
4b. Task-oriented coping	.08	.017	− .022	.067	10.78
4c. Avoidance-oriented coping	.04	.003	− .059	.021	1.95
Criterion = Task performance (R^2^= .148; F[11,74] = .979, p < .326^a^)					
1a. Planning and organizing	− .04	.014	− .102	.029	9.37
1b. Learning and memory	− .15	.006	− .109	.031	3.97
1c. Problem solving	− .06	.003	− .121	.022	2.30
1d. Adaptability	.16	.016	− .076	.060	10.65
1e. Concentration	.17	.032	− .043	.109	21.40
2. Conscientiousness	.34	.042	− .039	.133	28.75
3a. Persistency	.06	.016	− .080	.052	11.14
3b. Self-confidence	− .13	.005	− .120	.022	3.31
4a. Emotion-oriented coping	− .04	.004	− .105	.023	2.64
4b. Task-oriented coping	− .03	.007	− .105	.031	4.78
4c. Avoidance-oriented coping	− .00	.003	− .104	.024	1.68

*p < .05 **p < .01

^a^Results from multiple regression analysis using SPSS version 25

^b^Results form relative weight analysis using R

*b* unstandardized regression weight, *β* standardized regression weight, RW raw relative weight (within rounding error raw weights will sum to R^2^), CI-L lower bound of confidence interval used to test the statistical significance of raw weight, CI-U upper bound of confidence interval used to test the statistical significance of raw weight, RS-RW relative weight rescaled as a percentage of predicted variance in the criterion variable attributed to each predictor (within rounding error rescaled weights sum to 100%)

A somewhat different picture emerged when considering results of a multiple regression analysis in other-rating report. Here, other-rating reports of all 11 dimensions explained between 25% (work accuracy), 17% (work pace), and 16% (social behaviour at work) of variance in the respective aspects of work behaviour and 15% of variance in task performance. Owing to the intercorrelations between the 11 predictor dimensions, many individual beta-coefficients were not significant in the other-rating sample. Notable exceptions were concentration and conscientiousness, which were significant predictors of work accuracy. With this, H2 is also partly confirmed.

An examination of the relative weights analysis of the self-rating report revealed that persistency (RW = .08) made the strongest unique contribution to the dimension social behaviour at work. It explained a statistically significant amount of variance in social behaviour at work as for the tests of significance the 95% CIs did not contain zero.

Examination of the relative weights of the other-rating report, revealed that both concentration (RW = .06) and conscientiousness (RW = .06) made the strongest contribution to work accuracy. Next in the contribution to work accuracy came learning and memory (RW = .03) and persistency (RW = .03). Adaptability made the strongest contribution to work pace (RW = .04).

## Discussion

In this study, we aimed both to validate the predictive measures of the Maastricht Work Capacity Monitor (MW©M) of Van Ruitenbeek et al. [28], and to supplement this instrument with measures for work behaviour and task performance, in order to enhance human resource practices with respect to assessing the work capacity and the development of people with LWC.

We examined the relationship between the various personal characteristics and these two criterion variables (performance measures). Results showed corresponding significant correlations between the personal characteristics and the three dimensions of work behaviour and task performance in both self-report and other-rating report. Multiple regression analyses indicated in line with what we hypothesized, that avoidance-oriented coping was negatively related to all three work behaviour dimensions and to task performance in the self-report sample. Furthermore, corresponding to what we hypothesised, emotion-oriented coping was negatively related to work pace, and persistency was positively related to social behaviour at work. In the other-report sample, the expected positive relation between three predictors (learning and memory, concentration, and conscientiousness) and work accuracy was confirmed. However, in contrast with what we expected, self-confidence related negatively to work accuracy. This can be explained by the concern of practitioners that people from the target group tend to either over- (in this case) or underestimate themselves [69].

Although avoidance-oriented coping turned out to be the strongest predictor in multiple regression analyses, the relative weight analysis showed that persistency had the strongest unique contribution. This means that persistency has a stronger impact on behavioural outcomes than the tendency to walk away (avoidance-oriented coping), and that makes perfect sense.

Overall, results obtained with the self-rating and other-rating forms, showed a similar amount of variance explained by the models as a whole ranging from 14.8% to 24.7%. There were, however, some differences regarding the individual predictor-outcome relations between the self- and other-rating forms as well. This is in line with Connelly and Hülsheger [70]. These authors argue that research can benefit from collecting personality ratings from non-self-sources, such as significant others or other observers outside the work context. Our study also indicates that self- and other-rating perspectives are complementary. There is a strong tendency to look for self-other agreement in personality reports in literature [71], but we would like to make a case for ‘celebrating the differences’ in views. It is natural and functional that the ‘self-perspective’ differs from others’ perspective. Therefore, we think that science and practice can be enriched by research that focusses on underlying reasons for the differences in perspectives instead of searching for the similarities.

It is interesting to note that self-rated avoidance-oriented coping showed negative predictive validity, whereas self-rated task-oriented coping showed no significant predictive validity in multiple regression. In the other-rating sample, however, positive correlations were found between task-oriented coping and two work behaviour dimensions. These findings can be explained by findings in a meta-analytic comparison of self- and informant-report study of Kim and colleagues [71], which implied that people are generally accurate but somewhat self-effacing when rating their own personality traits.

Taken together, this study largely supported the validity of the MW©M in predicting work behaviour and task performance of people with LWC. It indicated that people with LWC are able to predict their own work behaviour accurately, similar to what is found in literature [71] regarding the general population.

### Practical Applications

This study contributes to practice as self- and other perspectives of personal characteristics are essential to getting a grip on how people function. The different perspectives can sharpen one’s view [70], so an instrument built upon multiple-source perspectives such as the extended MW©M, can enhance professional development of people with LWC. The fact that this study indicates that people with LWC are very well able to predict their work behaviour when scales are adapted is an important finding for professionals in the field (such as job coaches and vocational re-integration experts). These professionals can revise their persistent concern about the ability of people with LWC to reflect critically on their own behaviour and provide accurate responses. Professionals can now rely on validated measures that support talking with them instead of talking about them.

A noteworthy result is that self-rated avoidance-oriented coping stands out in negative predictive validity on all outcome variables. An explanation can be found in the fact that avoidance-oriented coping (e.g. looking for support from others, avoiding threat and searching security) is a natural coping response, whereas task-oriented coping (e.g. problem solving, and cognitive reframing or restructuring of a problem) is associated with more complex mental capacities [57]. It indicates that learned helplessness [72] is still a persistent phenomenon, and that the development of task-oriented coping gets too little attention. Apart from the above-mentioned practical implications, this indicates important directions for training, such as the call for practitioners to support people from the target group in the developing of task-oriented coping.

## Limitations and Future Research Directions

A strength of our design was that we included multi-source measurements at two time points. A weakness may be that this leads to many dropouts, but we still have sufficient power in this study.

With respect to future directions of research, we think that the development over time of the work capacity of people with LWC needs to be monitored. This is precisely the added value of this instrument. Furthermore, Tables 2 and 3 showed high correlations between all three dimensions of work behaviour, and task performance. It would be worthwhile to explore this relation more accurately for people with LWC since this corresponds to the literature, stating that in order to be able to deliver the expected performance, people need to display certain behaviour at work [35].

## Conclusion

To conclude, this study largely supports the predictive validity of the MW©M in predicting work behaviour and task performance. This study indicates that self- and other-rating perspectives are complementary to each other and it discusses the added value of research that focusses on the underlying reasons for disagreement between self- and other-report.

We argue that the extended MW©M enables adequate and fair performance evaluations of this specific population and contributes to science, by exploring the relationship between personality traits and work performance more accurately.

Moreover, this study contributes to vocational practices as it helps the continuous and methodological monitoring principle of supported employment interventions such as IPS [29] .

Finally, we argue that with the help of this instrument, our target group can learn to reflect on their own strengths and weaknesses. This enhances their personal and professional development and increases the chances for sustainable integration in work.

## Footnotes

1 Items for the other-rating forms are formulated in the third person.
